# Dataset of Comprehensive Thermal Performance on Cooling the Hot Tube Surfaces of Vortex Tube at Different Pressure and Fraction

**DOI:** 10.1016/j.dib.2020.105611

**Published:** 2020-04-22

**Authors:** Alfan Sarifudin, Danar Susilo Wijayanto, Indah Widiastuti, Nugroho Agung Pambudi

**Affiliations:** Department of Mechanical Engineering Education, Universitas Sebelas Maret, Sutami No.36 A Road, Pucangsawit, Jebres, Surakarta City, Central Java Province, Zip Code 57126, Indonesia

**Keywords:** Coefficient of the performance (COP), experiment, heat flow, isentropic efficiency, mathematical analysis

## Abstract

The performance of the vortex tube is low compared to a conventional heat pump engine based on Freon refrigerants, and therefore, there is a need for an experiment on how to improve its efficiency. This data article aims to analyze the effect of the new vortex tube design on cold temperature (*T_c_*), hot temperature (*T_h_*), delta cold temperature (Δ*T_c_*), delta hot temperature (Δ*T_h_*), heat transferred as cooling effect $\left({\dot{Q}}_{c}\right)$, heat transferred as heating effect $\left({\dot{Q}}_{h}\right)$, isentropic efficiency as cooling effect (*η*_*isc*_), isentropic efficiency as heating effect (*η*_*isc*_), the coefficient of performance refrigeration (*COP_ref_*), and coefficient of performance heat pump (*COP_h_*), which is tested based on pressure and fraction variations. The data were obtained from the experimental measurements. Data were collected at conditions with temperature controlled at 27 ± 0.1°C. All measuring instruments were supposed to be consistent for at least 5 min for data to be collected, although retrieval was conducted 4 times.

Specifications Table

**Table:** 

Subject	Engineering, Mechanical Engineering
Specific subject area	Heat transfer, Fluid dynamics, Thermodynamics, Heat and mass transfer, Thermophysical property measurement, Cryogenics, Counter-flow Ranque-Hilsch Vortex Tube, Heat Pump.
Type of data	Table Image Figure
How data were acquired	Data were collected from the experimental measurements and mathematical calculations. Measuring instruments used include pressure gauge, flowmeter (air), anemometer, thermocouple, flowmeter (water), and data acquisition. For mathematical calculations using a personal computer with Microsoft Office Excel software.
Data format	Raw Data
Parameters for data collection	The parameters for experimental data include the temperature of the air to the inlet, room air, air from hot and cold outlets, and water to the cooling tube. It also included the volume of the airflow rate to the inlet, the pressure of the air entering the channel to the inlet, maximum air velocity from cold outlet, testing air velocity of n^th^-testing from cold outlet, and cooling water flow rate volume.
Description of data collection	Data were collected based on the conditions of the test chamber, whose temperature was controlled at 27 ± 0.1°C. The data were taken in case all measuring instruments were consistent at least 5 minutes. Data Retrieval was carried out 4 times.
Data source location	Department of Mechanical Engineering Education, Universitas Sebelas Maret City/Town/Region: Central Java Province Country: Indonesia
Data accessibility	With the article
Related research article	Sarifudin, A., Wijayanto, D.S., Widiastuti, I, Parameters optimization of tube type, pressure, and mass fraction on vortex tube performance using the Taguchi method. International Journal of Heat and Technology, https://doi.org/10.18280/ijht.370230

## Value of the data

• The data describes comprehensive RHVT thermal performance on the new vortex tube design tested based on pressure and fraction variations.
• The data illustrates the design specifications of the new vortex tube design to improve their performance.
• The data describes the installation procedures and working specifications of the measuring instruments to determine the performance on the vortex tube.
• The data provides calculation procedures for mathematical analysis of the experimental measurement.

## 1 Data Description

The performance of the vortex tube is low compared to a conventional heat pump engine based on Freon refrigerants, and therefore, there is a need for an experiment on how to improve its efficiency. Furthermore, the vortex tube has several benefits, including no moving parts or mechanical wear, saving maintenance costs, no Freon use, and it is environmentally friendly [1,2]. According to previous studies, the parameters that might improve its performance include mass fraction, air pressure entering the inlet, material type, and geometry [3], [4], [5], [6], [7]. This experiment, therefore, aims to determine the best vortex tube performance parameters tested under variations in design, pressure and fraction. The performance dataset presented includes cold temperature (*T_c_*), hot temperature (*T_h_*), delta cold temperature (Δ*T_c_*), delta hot temperature(Δ*T_h_*), heat transferred as cooling effect $\left({\dot{Q}}_{c}\right)$, heat transferred a heating effect $\left({\dot{Q}}_{h}\right)$, isentropic efficiency as cooling effect (*η*_*isc*_), isentropic efficiency as heating effect (*η*_*isc*_), coefficient of performance refrigeration (*COP_ref_*), and coefficient of performance heat pump (*COP_h_*).

The average temperature of cold air produced by vortex tubes with natural cooling tube types is presented in Table 1 while Table 2 shows the vortex tubes with forced cooling. Temperature data are presented using °C units.

**Table 1:** Temperature average air exits from the natural cooling vortex tube cold outlet.

Cold air mass fraction	Air pressure to inlet		
	0.5bar	1.0bar	1.5bar
30%	21.650°C	18.250°C	15.850°C
40%	21.100°C	17.350°C	14.925°C
50%	21.900°C	18.650°C	16.375°C
60%	22.350°C	19.375°C	17.300°C
70%	22.825°C	20.175°C	18.350°C

**Table 2:** Temperature of the mean air exit from the vortex tube cold outlet and forced cooling.

Cold air mass fraction	Air pressure to inlet		
	0.5bar	1.0bar	1.5bar
30%	20.600°C	16.600°C	13.950°C
40%	20.200°C	16.050°C	13.450°C
50%	20.400°C	16.450°C	14.000°C
60%	20.600°C	16.850°C	14.575°C
70%	20.975°C	17.675°C	15.925°C

The average temperature of hot air produced by vortex tubes with natural cooling tube types is presented in Table 3, while Table 4 shows the vortex tubes with forced cooling. Temperature data are presented using °C units.

**Table 3:** Temperature average air exits from the natural cooling vortex tube heat outlet.

Cold air mass fraction	Air pressure to inlet		
	0.5bar	1.0bar	1.5bar
30%	29.300°C	30.075°C	30.500°C
40%	30.550°C	31.800°C	32.550°C
50%	32.500°C	35.050°C	36.650°C
60%	34.900°C	38.975°C	41.000°C
70%	32.500°C	35.750°C	37.850°C

**Table 4:** Temperature of the mean air exit from the vortex tube heat outlet and forcible cooling.

Cold air mass fraction	Air pressure to inlet		
	0.5bar	1.0bar	1.5bar
30%	27.400°C	27.550°C	27.650°C
40%	28.025°C	28.575°C	28.975°C
50%	28.325°C	29.050°C	29.600°C
60%	28.575°C	29.550°C	30.250°C
70%	28.050°C	28.650°C	29.050°C

Changes in the cold (Δ*T_c_*) or hot air temperature (Δ*T_h_*) is the difference in inlet temperature (*T_i_*) to cold outlet temperature (*T_c_*) or hot outlet temperature (*T_h_*), as shown in equation [8]

(1) $$\Delta T_{c}=\;T_{i}-T_{c}$$

(2) $$\Delta T_{h}=\;T_{h}-T_{i}$$

The changes in the cold temperature (Δ*T_c_*) of cold air produced by vortex tubes with natural cooling tube types is presented in Table 5 while Table 6 shows the vortex tubes with forced cooling. Temperature data are presented using °C units.

**Table 5:** Average changes in the cold temperature of air coming out of the natural cooling vortex tube cooling outlet.

Cold air mass fraction	Air pressure to inlet		
	0.5bar	1.0bar	1.5bar
30%	5.350°C	8.750°C	11.150°C
40%	5.900°C	9.650°C	12.075°C
50%	5.100°C	8.350°C	10.625°C
60%	4.650°C	7.625°C	9.700°C
70%	4.175°C	6.825°C	8.650°C

**Table 6:** Changes in the cold temperature mean air exits from the vortex tube cold forced cooling outlet.

Cold air mass fraction	Air pressure to inlet		
	0.5bar	1.0bar	1.5bar
30%	6.400°C	10.400°C	13.050°C
40%	6.800°C	10.950°C	13.550°C
50%	6.600°C	10.550°C	13.000°C
60%	6.400°C	10.150°C	12.425°C
70%	6.025°C	9.325°C	11.075°C

The hot air temperature (Δ*T_h_*) of hot air produced by vortex tubes with natural cooling tube types is presented in Table 7 while Table 8 shows the vortex tubes with forced cooling. Temperature data are presented using °C units.

**Table 7:** Changes temperature average air exits from the natural cooling vortex tube heat outlet.

Cold air mass fraction	Air pressure to inlet		
	0.5bar	1.0bar	1.5bar
30%	2.300°C	3.075°C	3.500°C
40%	3.550°C	4.800°C	5.550°C
50%	5.500°C	8.050°C	9.650°C
60%	7.900°C	11.975°C	14.000°C
70%	5.500°C	8.750°C	10.850°C

**Table 8:** Changes temperature of the mean air exit from the vortex tube heat outlet and forcible cooling.

Cold air mass fraction	Air pressure to inlet		
	0.5bar	1.0bar	1.5bar
30%	0.400°C	0.550°C	0.650°C
40%	1.025°C	1.575°C	1.975°C
50%	1.325°C	2.050°C	2.600°C
60%	1.575°C	2.550°C	3.250°C
70%	1.050°C	1.650°C	2.050°C

The temperature change in the isentropic process (Δ*T_is_*) is calculated by the following equation [8]

(3) $$\Delta T_{is}=\;T_{i}\left(1-{\left(\frac{P_{a}}{P_{i}}\right)}^{\frac{\gamma -1}{\gamma }}\right)$$

Where the specific heat ratio (*γ*) is the specific heat at constant pressure (*Cp*) per specific heat at constant volume (*Cv*) [9]. Based on the Cengel table appendix 1 (2006) for this ambient experiment the constant pressure (*Cp*) is 1.007*kJ*/*kg.K* and constant volume (*Cv*) is 0.7180*kJ*/*kg.K* [9]:

(4) $$\gamma =\frac{Cp}{Cv}$$

Temperature change in the isentropic process (Δ*T_is_*) produced by vortex tubes with natural cooling and forced cooling tube types is presented in Table 9 shows the vortex tubes with forced cooling. Temperature data are presented using °C units.

**Table 9:** Temperature change in the isentropic process of air enter into of the vortex tube inlet.

Air pressure to inlet		
0.5bar	1.0bar	1.5bar
32.63°C	53.68°C	68.88°C

Isentropic Efficiency (*η*_*is*_) is the sum of the changes in the inlet to outlet temperature at each isentropic temperature change, as shown in the following equation [8]:

(5) $$\eta_{\mathrm{is}}=\frac{\Delta T}{\Delta T_{\mathrm{is}}}$$

The cold outlet isentropic efficiency (*η*_*isc*_) and hot outlet isentropic efficiency (*η*_*ish*_) equations are shown by the following:

(6) $$\eta_{isc}=\frac{T_{i}-T_{c}}{T_{i}\left(1-{\left(\frac{P_{a}}{P_{i}}\right)}^{\frac{\gamma -1}{\gamma }}\right)}$$

(7) $$\eta_{ish}=\frac{T_{h}-T_{i}}{T_{i}\left(1-{\left(\frac{P_{a}}{P_{i}}\right)}^{\frac{\gamma -1}{\gamma }}\right)}$$

The Cold Isentropic Efficiency (*η*_*isc*_) of cold air produced by vortex tubes with natural cooling tube types is presented in Table 10 while Table 11 shows the vortex tubes with forced cooling. An isentropic Efficiency is a dimensionless number, expressed as a percentage number.

**Table 10:** Average Isentropic Efficiency of air coming out of the natural cooling vortex tube cooling outlet.

Cold air mass fraction	Air pressure to inlet		
	0.5bar	1.0bar	1.5bar
30%	16.39%	16.30%	16.19%
40%	18.08%	17.98%	17.53%
50%	15.63%	15.56%	15.43%
60%	14.25%	14.21%	14.08%
70%	12.79%	12.71%	12.56%

**Table 11:** Isentropic Efficiency mean air exits from the vortex tube cold forced cooling outlet.

Cold air mass fraction	Air pressure to inlet		
	0.5bar	1.0bar	1.5bar
30%	19.61%	19.38%	18.95%
40%	20.84%	20.40%	19.67%
50%	20.22%	19.65%	18.87%
60%	19.61%	18.91%	18.04%
70%	18.46%	17.37%	16.08%

The Hot Isentropic Efficiency (*η*_*ish*_) of hot air produced by vortex tubes with natural cooling tube types is presented in Table 12 while Table 13 shows the vortex tubes with forced cooling.

**Table 12:** Hot Isentropic Efficiency exits from the natural cooling vortex tube heat outlet.

Cold air mass fraction	Air pressure to inlet		
	0.5bar	1.0bar	1.5bar
30%	7.05%	5.73%	5.08%
40%	10.88%	8.94%	8.06%
50%	16.85%	15.00%	14.01%
60%	24.21%	22.31%	20.33%
70%	16.85%	16.30%	15.75%

**Table 13:** Hot Isentropic Efficiency exits from the vortex tube heat outlet and forcible cooling.

Cold air mass fraction	Air pressure to inlet		
	0.5bar	1.0bar	1.5bar
30%	1.23%	1.02%	0.94%
40%	3.14%	2.93%	2.87%
50%	4.06%	3.82%	3.77%
60%	4.83%	4.75%	4.72%
70%	3.22%	3.07%	2.98%

The fraction (ɛ_*c*_) of the cold outlet was obtained from the speed of the mass flow of air coming out, specifically ${\dot{m}}_{outc}$ at each mass flow rate of air entering the inlet ${\dot{m}}_{in}$. In Eq. (8), each air mass flow was measured at the same diameter and air pressure to obtain a simplified Eq. (9). In general, where ${\vec{v}}_{cn}$ is the speed of air coming out at the cold outlet on n-variable tested and ${\vec{v}}_{cmax}$ is the maximum mass flow rate of air coming out of the cold outlet with the heat outlet tightly closed. This is meant to satisfy the law of mass balance, which states that the mass coming out of the system is the same as the mass entering the system. The formulas to adjust the size of the fraction are as follows [2,3]

(8) $$\varepsilon_{c}={\dot{m}}_{out\_c}/{\dot{m}}_{in}$$

(9) $$\varepsilon_{c}={\vec{v}}_{c\_n}/{\vec{v}}_{c\_max}$$

Maximum wind speed comes out of the cold outlet for each pressure is 0.5bar (8.4m/s), 1.0bar (11.2m/s), and 1.5bar (14.0m/s). The regulation of the air velocity for each fraction is carried out by playing the valve gap in the hot outlet, then presented in Table 14.

**Table 14:** Air velocity exits the cold outlet.

Cold air mass fraction	Air pressure to inlet		
	0.5bar	1.0bar	1.5bar
30%	2.5m/s	3.4m/s	4.2m/s
40%	3.4m/s	4.5m/s	5.6m/s
50%	4.2m/s	5.6m/s	7.0m/s
60%	5.0m/s	6.7m/s	8.4m/s
70%	5.9m/s	7.8m/s	9.8m/s

The value of the volume flow rate (${\dot{V}}_{in}$) for each pressure is presented in Table 15 and presented in units of $m^{3}\;/\;s$.

**Table 15:** The rate of volume flow to the inlet.

Air pressure to inlet		
0.5bar	1.0bar	1.5bar
0.137029395$m^{3}\;/\;s$	0.252419435$m^{3}\;/\;s$	0.420141149$m^{3}\;/\;s$

Based on the Cengel table appendix 1 (2006) the value of gas constant (*R*) is 0.2870*kJ*/*kg.K* [9]. The gas density (*ρ*) equation is described as;

(10) $$\rho =P_{in\_absolute}/R\;T$$

Ambient gas temperature, used in Kelvin units, is 300.15K. The value of ρ in the following Table 16 is presented in units of $kg\;/\;m^{3}$.

**Table 16:** The density flow to the inlet.

Air pressure to inlet		
0.5bar	1.0bar	1.5bar
$P_{in\_absolute}$		
(151.335*Pa*)	(201.335*Pa*)	(251.335*Pa*)
Gas Density		
0.137029395$kg\;/\;m^{3}$	0.252419435$kg\;/\;m^{3}$	0.420141149$kg\;/\;m^{3}$

The mass flow rate flowing at the hot outlet is denoted as$\;{\dot{m}}_{outh}$. The *Cp* value can be determined by reading the table while T is the temperature of the air coming into through the hot outlet.

According to the equilibrium equation, the mass flow rate formula is as follows [2,10]

(11) $${\dot{m}}_{in}={\dot{m}}_{outc}+{\dot{m}}_{outh}$$

Where ${\dot{m}}_{in}$ is the air mass flow entering, while ${\dot{m}}_{outh}$ is the air mass flow coming out through the hot outlet. From Eq. (5), these variables are proportional in the inlet vortex tube. Since the instrument can only measure air conditions atthe inlet and the cold outlet, ${\dot{m}}_{outh}$ could be determined through the use of the equation.

The value of the incoming mass flow on the vortex tube is [2]

(12) $${\dot{m}}_{in}=\;{\dot{V}}_{in}\;\rho$$

The mass flow that enters the inlet (${\dot{m}}_{in}$) for each pressure in the Table 17 is presented in units of kg/s.

**Table 17:** Mass flow to the inlet.

Air pressure to inlet		
0.5bar	1.0bar	1.5bar
0.137kg/s	0.252kg/s	0.420kg/s

Mass flow at cold outlet (${\dot{m}}_{outc\_n}$) that occur at the n^th^ cold fraction $\left(\varepsilon_{c_{n}}\right)$ is denoted by the following equation:

(13) $${\dot{m}}_{outc\_n}=\;{\dot{m}}_{in}\times \varepsilon_{c\_n}$$

Mass flow out through the cold outlet in the Table 18 ${\dot{m}}_{outc}\;$is presented in the following table in unit kg/s.

**Table 18:** Mass flow exits from the cold outlet.

Cold air mass fraction	Air pressure to inlet		
	0.5bar	1.0bar	1.5bar
30%	0.041kg/s	0.076kg/s	0.126kg/s
40%	0.055kg/s	0.101kg/s	0.168kg/s
50%	0.069kg/s	0.126kg/s	0.210kg/s
60%	0.082kg/s	0.151kg/s	0.252kg/s
70%	0.096kg/s	0.177kg/s	0.294kg/s

Refer to Eq. 11, the mass flow through the hot outlet (${\dot{m}}_{outh\_n}$) that occur at the n^th^ cold fraction $\left(\varepsilon_{c_{n}}\right)$ is denoted by the following equation:

(14) $${\dot{m}}_{outh\_n}=\;{\dot{m}}_{in}-{\dot{m}}_{outh\_n}$$

Mass flow out through the hot outlet ${\dot{m}}_{outh}\;$is presented in the Table 19 in unit kg/s.

**Table 19:** Mass flow exits from the hot outlet.

Cold air mass fraction	Air pressure to inlet		
	0.5bar	1.0bar	1.5bar
30%	0.096kg/s	0.177kg/s	0.294kg/s
40%	0.082kg/s	0.151kg/s	0.252kg/s
50%	0.069kg/s	0.126kg/s	0.210kg/s
60%	0.055kg/s	0.101kg/s	0.168kg/s
70%	0.041kg/s	0.076kg/s	0.126kg/s

The amount of heat that can be transferred by the vortex tube as a cooling effect is denoted as ${\dot{Q}}_{c}$. It is obtained using the following equation [2,^3^,^11^,12]:

(15) $${\dot{Q}}_{c}={\dot{m}}_{outc}\;Cp\;\left(T_{c}-T_{in}\right)$$

Where ${\dot{m}}_{outc}$ is the air mass flowing every second at the cold outlet, *Cp* is the capacity of air at ambient during the test, *T_c_* is the value of the air temperature through the cold outlet, and *T_in_* is the temperature of the air entering the inlet vortex tube. The *T_c_* and *T_in_* values were obtained from the data measured.

The Table 20 dan Table 21 present the heat flow rate for cold outlet in kJ/s.

**Table 20:** Heat flow rate at cold outlet on natural cooling RHVT.

Cold air mass fraction	Air pressure to inlet		
	0.5bar	1.0bar	1.5bar
30%	0.221kJ/s	0.667kJ/s	1.415kJ/s
40%	0.326kJ/s	0.981kJ/s	2.043kJ/s
50%	0.352kJ/s	1.061kJ/s	2.248kJ/s
60%	0.385kJ/s	1.163kJ/s	2.462kJ/s
70%	0.403kJ/s	1.214kJ/s	2.562kJ/s

**Table 21:** Heat flow rate at cold outlet on force cooling RHVT.

Cold air mass fraction	Air pressure to inlet		
	0.5bar	1.0bar	1.5bar
30%	0.265kJ/s	0.793kJ/s	1.656kJ/s
40%	0.375kJ/s	1.113kJ/s	2.293kJ/s
50%	0.455kJ/s	1.341kJ/s	2.750kJ/s
60%	0.530kJ/s	1.548kJ/s	3.154kJ/s
70%	0.582kJ/s	1.659kJ/s	3.280kJ/s

The amount of heat transferred by the vortex tube as a heating effect is denoted by ${\dot{Q}}_{h}$ and obtained using the following equation [13]:

(16) $${\dot{Q}}_{h}={\dot{m}}_{outh}\;Cp\;\left(T_{h}-T_{in}\right)$$

The mass flow rate flowing at the hot outlet is denoted as$\;{\dot{m}}_{outh}$. The *Cp* value can be determined by reading the table while T is the temperature of the air coming into through the hot outlet. The Table 22 and Table 23 present the heat flow rate for hot outlet in kJ/s.

**Table 22:** Heat flow rate at hot outlet on natural cooling RHVT.

Cold air mass fraction	Air pressure to inlet		
	0.5bar	1.0bar	1.5bar
30%	0.222kJ/s	0.547kJ/s	1.037kJ/s
40%	0.294kJ/s	0.732kJ/s	1.409kJ/s
50%	0.379kJ/s	1.023kJ/s	2.041kJ/s
60%	0.436kJ/s	1.218kJ/s	2.369kJ/s
70%	0.228kJ/s	0.667kJ/s	1.377kJ/s

**Table 23:** Heat flow rate at hot outlet on force cooling RHVT.

Cold air mass fraction	Air pressure to inlet		
	0.5bar	1.0bar	1.5bar
30%	0.039kJ/s	0.098kJ/s	0.193kJ/s
40%	0.085kJ/s	0.240kJ/s	0.501kJ/s
50%	0.091kJ/s	0.261kJ/s	0.550kJ/s
60%	0.087kJ/s	0.259kJ/s	0.550kJ/s
70%	0.043kJ/s	0.126kJ/s	0.260kJ/s

The total compressed air power entering the inlet with ideal isothermal compression is as follows [3,^6^,^7^,[13], [14], [15]:

(17) $$W={\dot{m}}_{in}\;RT_{in}\;ln\;\left(P_{in}/P_{atm}\right)$$

From Eq. (17), ${\dot{m}}_{in}$ is the mass flow rate of air entering the inlet channel, *R* is the specific gas constant, *T_in_* is the temperature of the air, and *P_in_* is the air pressure entering the inlet channel. The environmental air pressure is denoted by *P_atm_*. The Table 24 presents the total compressed air power entering the inlet in kJ/s.

**Table 24:** Total compressed air power entering the inlet.

Air pressure to inlet		
0.5bar	1.0bar	1.5bar
4.734kJ/s	14.928kJ/s	32.875kJ/s

The coefficient of performance refrigeration (COP_ref_) is a dimensionless number that measures the performance of a cooling heat pump engine when transferring heat from a cooled room [2,12]. For a vortex tube, it is calculated as follows [2,^3^,12].

(18) $$COP_{ref}={\dot{Q}}_{c}/\dot{W}$$

The average COP_ref_ produced by vortex tube with natural cooling tube type is presented in Table 25 while the vortex tube with forced cooling is shown in Table 26. COP_ref_ numbers are dimensionless, therefore they are presented without units.

**Table 25:** COP_ref_ average air exits from the natural vortex tube cold cooling outlet.

Cold air mass fraction	Air pressure to inlet		
	0.5bar	1.0bar	1.5bar
30%	0.047	0.046	0.044
40%	0.069	0.068	0.062
50%	0.074	0.074	0.070
60%	0.081	0.081	0.079
70%	0.085	0.083	0.083

**Table 26:** COP_ref_ the mean air coming out of the vortex cold tube forced cooling outlet.

Cold air mass fraction	Air pressure to inlet		
	0.5bar	1.0bar	1.5bar
30%	0.056	0.054	0.050
40%	0.079	0.078	0.069
50%	0.096	0.093	0.087
60%	0.112	0.104	0.097
70%	0.123	0.111	0.100

The coefficient of performance heat pumps (COP_h_) is a dimensionless number that measures the performance of a heat pump engine when transferring heat to a heated chamber [2,12]. It was denoted as follows in the vortex tube [2,^3^,12].

(19) $$COP_{h}={\dot{Q}}_{h}/\dot{W}$$

Table 27 and 28 shows the average COP_h_ produced by vortex tubes with natural cooling tube types and vortex tubes with forced cooling respectively. There are no dimensions for COP_h_ numbers.

**Table 27:** COP_h_ mean air exit from the vortex tube and cold forced outlet.

Cold air mass fraction	Air pressure to inlet		
	0.5bar	1.0bar	1.5bar
30%	0.047	0.037	0.036
40%	0.066	0.049	0.043
50%	0.080	0.068	0.062
60%	0.076	0.081	0.072
70%	0.039	0.043	0.042

**Table 28:** COP_h_ average air exits from the vortex tube cold forced outlet.

Cold air mass fraction	Air pressure to inlet		
	0.5bar	1.0bar	1.5bar
30%	0.008	0.007	0.006
40%	0.018	0.016	0.015
50%	0.019	0.017	0.017
60%	0.018	0.017	0.017
70%	0.009	0.008	0.008

**Table 29:** Experimental devices.

Initial	Name	Function	Symbol	Unit	Sensitivity values
AC	Air Conditioner	Room temperature controller	-	°Celsius	0.1
C	Compressor	Pressurized air supply	-	-	-
AT	Air Tank	Maintained air pressure so as not to drop, reduce humidity and return to room temperature	-	-	-
PR	Pressure Regulator	Sets the input air pressure	-	-	-
PG	Pressure Gauge	Measured inlet air pressure	*P_in_*	bar	0.1
AFM	Flowmeter (air)	Measured airflow	${\dot{V}}_{in}$	Liter/min	0.1
VTA / VTB	Vortex tube (type A and type B)	Test instrument	-	-	-
AM	Anemometer	Measured the speed of cold outlet air	${\vec{v}}_{cn}$ and ${\vec{v}}_{cmax}$	meter/sec	0.1
TCD	Thermocouple Display	Measured hot and cold outlet air temperature	−	°Celsius	0.01
WCS	Water Container Storage	Saved water for cooling	-	-	-
WCT	Water Container Trash	Saved waste water for cooling	-	-	-
P	Pump	Pumped cooling water	-	-	-
WFM	Flowmeter (water)	Measured water discharge	${\dot{V}}_{water}$	Liter/min	0.1
DA	Data Acquisition	Recorded measurement data	-	-	-
PC	Personal Computer	Processed observation data from each measuring instrument	-	-	-
TCS1	Thermocouple Sensor 1 (Type K)	Measured the temperature of the inlet air	*T_in_*	°Celsius	-
TCS2	Thermocouple Sensor 2 (Type K)	Measured the temperature of the cold outlet air	*T_c_*	°Celsius	-
TCS3	Thermocouple Sensor 3 (Type K)	Measured the temperature of the hot outlet air	*T_h_*	°Celsius	-
TCS4	Thermocouple Sensor 4 (Type K)	Measured ambient air temperature	T	°Celsius	-
TCS5	Thermocouple Sensor 5 (Type K)	Measured the temperature of the cooling water air	-	°Celsius	-

## 2 Experimental Design, Materials, and Methods

**Figure 1:**
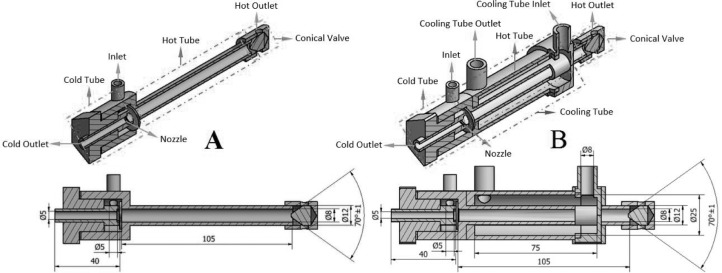
Two types of vortex tubes, A and B.

Data collection was carried out through experimental tests and processed mathematically. The RHVTs include types A with a natural cooling process and B with the forced cooling process, both on the surfaces of the tube. The RHVT used in types A and B was counter flow and the material used in all case was aluminium, except the cooling tube of B which used the black Teflon. The inlet diameter was 5mm, while the cold and hot tube had diameters and lengths 5mm and 40mm as well as 8mm and 105mm respectively. The diameter of the inlet and outlet of the cooling tube on type B RHVT was 8mm. However, an inner tube had a diameter of 25mm and a length of 75mm. Fig. 1 shows the details of the RHVT specifications used.

**Figure 2:**
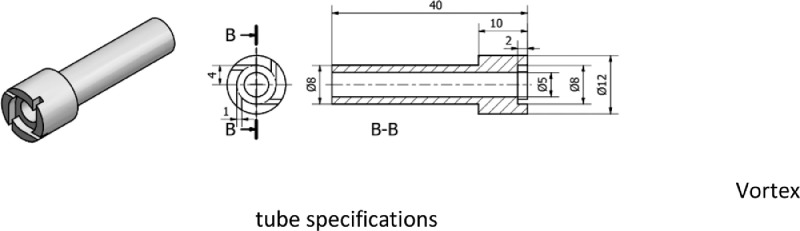
RHVT contra flow nozzles specifications.

Types A and B had 4 nozzle holes and the air rotation is in the direction of the flow at the inlet. The nozzle hole is rectangular with dimensions of 1 × 2mm. The diameter of the round nozzles is 8mm with a thickness of 1.5mm. The detailed specifications of the RHVT contra flow nozzles used are presented in Fig. 2.

**Figure 3:**
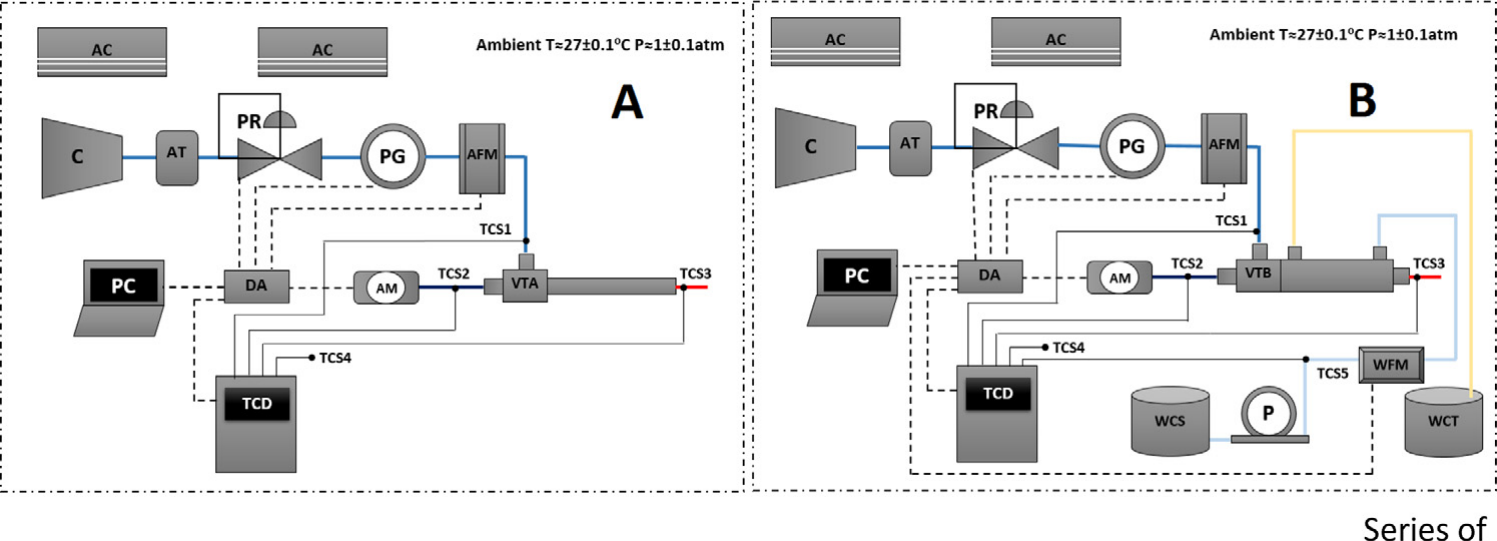
Series of experimental tools for types A and B.

Fig. 3 shows a series of instruments used for experimental data collection. In the Left and right sides of the figure are Type A and B vortex tubes respectively. The temperature was set at 27^o^±0.1°C and data were recorded after 5 minutes of running.

## Appendix Supplementary materials

Image, application 1

Image, application 2
