# Supplementary material for: Dataset of Comprehensive Thermal Performance on Cooling the Hot Tube Surfaces of Vortex Tube at Different Pressure and Fraction
Source: Data Brief. 2020 Apr 22;30:105611. doi: 10.1016/j.dib.2020.105611 (PMC7210404; doi:10.1016/j.dib.2020.105611)
Supplement: Supplementary file 2 [file mmc2.pdf]

DATASET OF COMPREHENSIVE THERMAL PERFORMANCE ON COOLING THE HOT TUBE SURFACES OF VORTEX TUBE AT DIFFERENT PRESSURE AND FRACTION

by Nugroho Agung Pambudi

Submission date: 11-Apr-2020 12:29PM (UTC+0700)
Submission ID: 1295012925
File name: Manuscript_REVISED.docx (442.28K)
Word count: 4246
Character count: 21950

Article Title

DATASET OF COMPREHENSIVE THERMAL PERFORMANCE ON COOLING THE HOT TUBE SURFACES OF VORTEX TUBE AT DIFFERENT PRESSURE AND FRACTION

Authors

Alfan Sarifudin[1], Danar Susilo Wijayanto[1], Indah Widiastuti[1], Nugroho Agung Pambudi[1]

Affiliations

1. *Department of Mechanical Engineering Education, Faculty of Teacher Training and Education, Universitas Sebelas Maret*

Corresponding author(s)

Alfan Sarifudin (alfansarifudin.indonesia@gmail.com; alfansarifudin@merahputih.id)

Abstract

The performance of the vortex tube is low compared to a conventional heat pump engine based on Freon refrigerants, and therefore, there is a need for a experiment on how to improve its efficiency. This data article aims to analyse the effect of the new vortex tube design on temperature (T_c), hot temperature (T_h), delta cold temperature (ΔT_c), delta hot temperature (ΔT_h), heat transferred as cooling effect $(\dot{Q}_c)$, heat transferred a heating effect $(\dot{Q}_h)$, isentropic efficiency as cooling effect (η_{isc}), isentropic efficiency as heating effect (η_{isc}), coefficient of performance refrigeration (COP_{ref}), and coefficient of performance heat pump (COP_h), which is tested based on pressure and fraction variations. The data was obtained from the experimental measurements. Data was collected at conditions with temperature controlled at at 27 ± 0.1°C. All measuring instruments were supposed to be consistent for at least 5 minutes for data to be collected, though retrieval was conducted 4 times.

Keywords

coefficient of the performance (COP); experiment; heat flow; isentropic efficiency; mathematical analysis.

Specifications Table

**Subject**	Engineering, Mechanical Engineering
**Specific subject area**	Heat transfer, Fluid dynamics, Thermodynamics, Heat and mass transfer, Thermophysical property measurement, Cryogenics, Counter-flow Ranque-Hilsch Vortex Tube, Heat Pump.
**Type of data**	Table<br>Image

	Graph<br>Figure
**How data were acquired**	Data were collected from the experimental measurements and mathematical calculations. Measuring instruments used include Digital thermometer, Dew point meter, Flowmeter to air, Pressure Gauge, Thermocouple, Thermometer Anemometer, Flowmeter, and measuring cup. For mathematical calculations, a personal computer with Microsoft Office Excel software and an online calculator were used to determine the air density.
**Data format**	Raw Data
**Parameters for data collection**	The parameters for experimental data include the temperature of the air to the inlet, room air, air from hot and cold outlets, water to the cooling tube, and air dew point. It also included the volume of the airflow rate to the inlet, the pressure of the air entering the channel to the inlet, maximum air velocity from cold outlets, testing air velocity of nth-testing from cold outlets, and cooling water flow rate volume.
**Description of data collection**	Data were collected based on the conditions of the test chamber, whose temperature was controlled at 27 ± 0.1ºC. The data were taken in case all measuring instruments were consistent at least 5 minutes. Data Retrieval was carried out 4 times.
**Data source location**	Department of Mechanical Engineering Education, Universitas Sebelas Maret<br>City/Town/Region: Central Java Province<br>Country: Indonesia
**Data accessibility**	With the article
**Related research article**	Sarifudin, A., Wijayanto, D.S., Widiastuti, I, Parameters optimization of tube type, pressure, and mass fraction on vortex tube performance using [illegible]e Taguchi method. International Journal of Heat and Technology, https://doi.org/10.18280/ijht.370230

Value of the Data

- The data describes comprehensive RHVT thermal performance on the new vortex tube design tested based on pressure and fraction variations.

- The data illustrates the design specifications of the new vortex tube design to improve their performance.
- The data describes the installation procedures and working specifications of the measuring instruments to determine the performance on the vortex tube.
- The data provides calculation procedures for mathematical analysis of the experimental measurement.

Data Description

The performance of the vortex tube is low compared to a conventional heat pump engine based on Freon refrigerants, and therefore, there is a need for a experiment on how to improve its efficiency. Furthermore, the vortex tube has several benefits, including no moving parts or mechanical wear, saving maintenance costs, no Freon use, and it is environmentally friendly [1], [2]. According to previous studies, the parameters that might improve its performance include mass fraction, air pressure entering the inlet, material type, and geometry [3]–[7]. This experiment, therefore, aims to determine the best vortex tube performance parameters tested under variations in design, pressure and fraction. The performance dataset presented includes cold temperature (T_c), hot temperature (T_h), delta cold temperature (ΔT_c), delta hot temperature(ΔT_h), heat transferred as cooling effect $(\dot{Q}_c)$, heat transferred a heating effect $(\dot{Q}_h)$, isentropic efficiency as cooling effect (η_{isc}), isentropic efficiency as heating effect (η_{isc}), coefficient of performance refrigeration (COP_{ref}), and coefficient of performance heat pump (COP_h).

The average temperature of cold air produced by vortex tubes with natural cooling tube types is presented in table 1 while table 2 shows the vortex tubes with forced cooling. Temperature data are presented using ^{o}C units.

Table 1. Temperature average air exits from the natural cooling vortex tube cold outlet

Cold air mass fraction	Air pressure to inlet		
	0.5bar	1.0bar	1.5bar
30%	21.650	18.250	15.850
40%	21.100	17.350	14.925
50%	21.900	18.650	16.375
60%	22.350	19.375	17.300
70%	22.825	20.175	18.350

Table 2. Temperature of the mean air exit from the vortex tube cold outlet and forced cooling

Cold air mass fraction	Air pressure to inlet		
	0.5bar	1.0bar	1.5bar
30%	20.600	16.600	13.950
40%	20.200	16.050	13.450
50%	20.400	16.450	14.000
60%	20.600	16.850	14.575

70%	20.975	17.675	15.925

The average temperature of hot air produced by vortex tubes with natural cooling tube types is presented in table 3 while table 4 shows the vortex tubes with forced cooling. Temperature data are presented using °C units.

Table 3. Temperature average air exits from the natural cooling vortex tube heat outlet

Cold air mass fraction	Air pressure to inlet		
	0.5bar	1.0bar	1.5bar
30%	29.300°C	30.075°C	30.500°C
40%	30.550°C	31.800°C	32.550°C
50%	32.500°C	35.050°C	36.650°C
60%	34.900°C	38.975°C	41.000°C
70%	32.500°C	35.750°C	37.850°C

5

Table 4. Temperature of the mean air exit from the vortex tube heat outlet and forcible cooling

Cold air mass fraction	Air pressure to inlet		
	0.5bar	1.0bar	1.5bar
30%	27.400°C	27.550°C	27.650°C
40%	28.025°C	28.575°C	28.975°C
50%	28.325°C	29.050°C	29.600°C
60%	28.575°C	29.550°C	30.250°C
70%	28.050°C	28.650°C	29.050°C

21

Changes in the cold (ΔT_c) or hot air temperature (ΔT_h) is the difference in inlet temperature (T_i) to cold outlet temperature (T_c) or hot outlet temperature (T_h), as shown in equation[8]

$$\Delta T_c = T_i - T_c \quad (1)$$

$$\Delta T_h = T_h - T_i \quad (2)$$

The changes in the cold temperature (ΔT_c) of cold air produced by vortex tubes with natural cooling tube types is presented in table 5 while table 6 shows the vortex tubes with forced cooling. Temperature data are presented using °C units.

Table 5. Average changes in the cold temperature of air coming out of the natural cooling vortex tube cooling outlet

Cold air mass fraction	Air pressure to inlet		
	0.5bar	1.0bar	1.5bar
30%	5.350°C	8.750°C	11.150°C
40%	5.900°C	9.650°C	12.075°C
50%	5.100°C	8.350°C	10.625°C

60%	4.650°C	7.625°C	9.700°C
70%	4.175°C	6.825°C	8.650°C

Table 6. Changes in the cold temperature mean air exits from the vortex tube cold forced cooling outlet

Cold air mass fraction	Air pressure to inlet		
	0.5bar	1.0bar	1.5bar
30%	6.400°C	10.400°C	13.050°C
40%	6.800°C	10.950°C	13.550°C
50%	6.600°C	10.550°C	13.000°C
60%	6.400°C	10.150°C	12.425°C
70%	6.025°C	9.325°C	11.075°C

The hot air temperature (ΔT_h) of hot air produced by vortex tubes with natural cooling tube types is presented in table 7 while table 8 shows the vortex tubes with forced cooling. Temperature data are presented using °C units.

Table 7. Changes temperature average air exits from the natural cooling vortex tube heat outlet

Cold air mass fraction	Air pressure to inlet		
	0.5bar	1.0bar	1.5bar
30%	2.300°C	3.075°C	3.500°C
40%	3.550°C	4.800°C	5.550°C
50%	5.500°C	8.050°C	9.650°C
60%	7.900°C	11.975°C	14.000°C
70%	5.500°C	8.750°C	10.850°C

Table 8. Changes temperature of the mean air exit from the vortex tube heat outlet and forcible cooling

Cold air mass fraction	Air pressure to inlet		
	0.5bar	1.0bar	1.5bar
30%	0.400°C	0.550°C	0.650°C
40%	1.025°C	1.575°C	1.975°C
50%	1.325°C	2.050°C	2.600°C
60%	1.575°C	2.550°C	3.250°C
70%	1.050°C	1.650°C	2.050°C

The temperature change in the isentropic process (ΔT_{is}) is calculated by the following equation [8]

$$\Delta T_{is} = T_i\left(1-\left(\frac{P_a}{P_i}\right)^{\frac{\gamma-1}{\gamma}}\right) \quad (3)$$

Where the specific heat ratio (γ) is the specific heat at constant pressure (Cp) per specific heat at constant volume (Cv)[9]. Based on the Cengel table appendix 1 (2006) for this ambient experiment the constant pressure (Cp) is $1.007\ kJ/kg.K$ and constant volume (Cv) is $0.7180 kJ/kg.K$ [9]:

$$\gamma = \frac{Cp}{Cv} \tag{4}$$

Temperature change in the isentropic process (ΔT_{is}) produced by vortex tubes with natural cooling and forced cooling tube types is presented in table 9 shows the vortex tubes with forced cooling. Temperature data are presented using °C units.

Table 9. Temperature change in the isentropic process of air enter into of the vortex tube inlet

Air pressure to inlet		
0.5bar	1.0bar	1.5bar
32.63°C	53.68°C	68.88°C

Isentropic Efficiency (η_{is}) is the sum of the changes in the inlet to outlet temperature at each isentropic temperature change, as shown in the following equation[8]:

$$\eta_{is} = \frac{\Delta T}{\Delta T_{isc}} \tag{5}$$

The cold outlet $\eta_{isc}$and hot η_{ish}outlet insontropic equations are shown by the following:

$$\eta_{isc} = \frac{T_i - T_c}{T_i\left(1 - \left(\frac{P_a}{P_i}\right)^{\frac{\gamma-1}{\gamma}}\right)} \tag{6}$$

$$\eta_{ish} = \frac{T_h - T_i}{T_i\left(1 - \left(\frac{P_a}{P_i}\right)^{\frac{\gamma-1}{\gamma}}\right)} \tag{7}$$

The Cold Isentropic Efficiency (η_{isc}) of cold air produced by vortex tubes with natural cooling tube types is presented in table 10 while table 11 shows the vortex tubes with forced cooling. An isentropic Efficiency is a dimensionless number, expressed as a percentage number.

Table 10. Average Isentropic Efficiency of air coming out of the natural cooling vortex tube cooling outlet

Cold air mass fraction	Air pressure to inlet		
	0.5bar	1.0bar	1.5bar
30%	16.39%	16.30%	16.19%
40%	18.08%	17.98%	17.53%
50%	15.63%	15.56%	15.43%
60%	14.25%	14.21%	14.08%

70%	12.79%	12.71%	12.56%

Table 11. Isentropic Efficiency mean air exits from the vortex tube cold forced cooling outlet

Cold air mass fraction	Air pressure to inlet		
	0.5bar	1.0bar	1.5bar
30%	19.61%	19.38%	18.95%
40%	20.84%	20.40%	19.67%
50%	20.22%	19.65%	18.87%
60%	19.61%	18.91%	18.04%
70%	18.46%	17.37%	16.08%

The Hot Isentropic Efficiency (η_{ish}) of hot air produced by vortex tubes with natural cooling tube types is presented in table 12 while table 13 shows the vortex tubes with forced cooling.

Table 12. Hot Isentropic Efficiency exits from the natural cooling vortex tube heat outlet

Cold air mass fraction	Air pressure to inlet		
	0.5bar	1.0bar	1.5bar
30%	7.05%	5.73%	5.08%
40%	10.88%	8.94%	8.06%
50%	16.85%	15.00%	14.01%
60%	24.21%	22.31%	20.33%
70%	16.85%	16.30%	15.75%

Table 13. Hot Isentropic Efficiency exits from the vortex tube heat outlet and forcible cooling

Cold air mass fraction	Air pressure to inlet		
	0.5bar	1.0bar	1.5bar
30%	1.23%	1.02%	0.94%
40%	3.14%	2.93%	2.87%
50%	4.06%	3.82%	3.77%
60%	4.83%	4.75%	4.72%
70%	3.22%	3.07%	2.98%

The fraction (ε_c) of the cold outlet was obtained from the speed of the mass flow of air coming out, specifically $\dot{m}_{outc}$ at each mass flow rate of air entering the inlet $\dot{m}_{in}$. In equation (8), each air mass flow was measured at the same diameter and air pressure to obtain a simplified equation (9). In general, where $\vec{v}_{cn}$ is the speed of air coming out at the cold outlet on n-variable tested and $\vec{v}_{cmax}$ is the maximum mass flow rate of air coming out of the cold outlet with the heat outlet tightly closed. This is meant to satisfy the law of mass balance, which states that the mass coming out of the system is the same as the mass entering the system. The formulas to adjust the size of the fraction are as follows [2], [3]

$$\varepsilon_c = \dot{m}_{out\_c}/\dot{m}_{in} \quad (8)$$

$\varepsilon_c = \vec{v}_{c\_n} / \vec{v}_{c\_max}$ (9)

Maximum wind speed comes out of the cold outlet for each pressure is 0.5bar (8.4$^m/_s$), 1.0bar (11.2$^m/_s$), and 1.5bar (14.0$^m/_s$). The regulation of the air velocity for each fraction is carried out by playing the valve gap in the hot outlet, then presented in table 14 below:

Table 14. Air velocity exits the cold outlet

Cold air mass fraction	Air pressure to inlet		
	0.5bar	1.0bar	1.5bar
30%	2.5$^m/_s$	3.4$^m/_s$	4.2$^m/_s$
40%	3.4$^m/_s$	4.5$^m/_s$	5.6$^m/_s$
50%	4.2$^m/_s$	5.6$^m/_s$	7.0$^m/_s$
60%	5.0$^m/_s$	6.7$^m/_s$	8.4$^m/_s$
70%	5.9$^m/_s$	7.8$^m/_s$	9.8$^m/_s$

The value of the volume flow rate ($\dot{V}_{in}$) for each pressure is presented in table 15 and presented in units of $^{m^3}/_s$.

Table 15. The rate of volume flow to the inlet

Air pressure to inlet		
0.5bar	1.0bar	1.5bar
0.137029395$^{\boldsymbol{m^3}}/_{\boldsymbol{s}}$	0.252419435$^{m^3}/_s$	0.420141149$^{m^3}/_s$

Based on the Cengel table appendix 1 (2006) the value of gas constant (R) is 0.2870 $kJ/kg.K$ [9] . The gas density (ρ) equation is described as;

$\rho = \mathrm{P}_{in\_absolute} / R\ \mathrm{T}$ (10)

Ambient gas temperature, used in Kelvin units, is 300.15K. The value of ρ in the following table 16 is presented in units of $^{kg}/_{m^3}$.

Table 16. The density flow to the inlet

Air pressure to inlet		
0.5bar	1.0bar	1.5bar
$\mathbf{P}_{in\_absolute}$		
(151.335$\boldsymbol{Pa}$)	(201.335Pa)	(251.335Pa)
Gas Density		
0.137029395$^{\boldsymbol{kg}}/_{\boldsymbol{m^3}}$	0.252419435$^{kg}/_{m^3}$	0.420141149$^{kg}/_{m^3}$

The mass flow rate flowing at the hot outlet is denoted as $\dot{m}_{outh}$. The Cp value can be determined by reading the table while T is the temperature of the air coming into through the hot outlet.

According to the equilibrium equation, the mass flow rate formula is as follows[2], [10]

$$\dot{m}_{in} = \dot{m}_{outc} + \dot{m}_{outh} \tag{11}$$

Where $\dot{m}_{in}$ is the air mass flow entering, while $\dot{m}_{outh}$ is the air mass flow coming out through the hot outlet. From equation (5), these variables are proportional in the inlet vortex tube. Since the instrument can only measure air conditions at cold inlets and outlets, $\dot{m}_{outh}$ could be determined through the use of the equation.

The value of the incoming mass flow on the vortex tube is [2]

$$\dot{m}_{in} = \dot{V}_{in}\,\rho \tag{12}$$

The mass flow that enters the inlet ($\dot{m}_{in}$) for each pressure is as follows presented in units of $^{kg}/_{s}$.

Table 17. Mass flow to the inlet

Air pressure to inlet		
0.5bar	1.0bar	1.5bar
0.137$^{\boldsymbol{kg}}/_{\boldsymbol{s}}$	0.252$^{kg}/_{s}$	0.420$^{kg}/_{s}$

Mass flow at cold outlets ($\dot{m}_{outc\_n}$) that occur at the nth cold fraction (ε_{c_n}) is denoted by the following equation:

$$\dot{m}_{outc\_n} = \dot{m}_{in} \times \varepsilon_{c\_n} \tag{13}$$

Mass flow out through the cold outlet $\dot{m}_{outc}$ is presented in the following table in unit $^{kg}/_{s}$:

Table 18. Mass flow exits from the cold outlet

Cold air mass fraction	Air pressure to inlet		
	0.5bar	1.0bar	1.5bar
30%	0.041$^{kg}/_{s}$	0.076$^{kg}/_{s}$	0.126$^{kg}/_{s}$
40%	0.055$^{kg}/_{s}$	0.101$^{kg}/_{s}$	0.168$^{kg}/_{s}$
50%	0.069$^{kg}/_{s}$	0.126$^{kg}/_{s}$	0.210$^{kg}/_{s}$
60%	0.082$^{kg}/_{s}$	0.151$^{kg}/_{s}$	0.252$^{kg}/_{s}$
70%	0.096$^{kg}/_{s}$	0.177$^{kg}/_{s}$	0.294$^{kg}/_{s}$

Refer to equation 11, the mass flow through the hot outlet ($\dot{m}_{outh\_n}$) that occur at the nth cold fraction (ε_{c_n}) is denoted by the following equation:

$$\dot{m}_{outh\_n} = \dot{m}_{in} - \dot{m}_{outh\_n} \tag{14}$$

Mass flow out through the hot outlet $\dot{m}_{outh}$ is presented in the following table in unit ${}^{kg}/_{s}$:

Table 19. Mass flow exits from the hot outlet

Cold air mass fraction	Air pressure to inlet		
	0.5bar	1.0bar	1.5bar
30%	$0.096\,{}^{kg}/_{s}$	$0.177\,{}^{kg}/_{s}$	$0.294\,{}^{kg}/_{s}$
40%	$0.082\,{}^{kg}/_{s}$	$0.151\,{}^{kg}/_{s}$	$0.252\,{}^{kg}/_{s}$
50%	$0.069\,{}^{kg}/_{s}$	$0.126\,{}^{kg}/_{s}$	$0.210\,{}^{kg}/_{s}$
60%	$0.055\,{}^{kg}/_{s}$	$0.101\,{}^{kg}/_{s}$	$0.168\,{}^{kg}/_{s}$
70%	$0.041\,{}^{kg}/_{s}$	$0.076\,{}^{kg}/_{s}$	$0.126\,{}^{kg}/_{s}$

The amount of heat that can be transferred by the vortex tube as a cooling effect is denoted as $\dot{Q}_c$. It is obtained using the following equation [2], [3], [11], [12]:

$$\dot{Q}_c = \dot{m}_{outc}\, Cp\, (T_c - T_{in}) \tag{15}$$

Where $\dot{m}_{outc}$ is the air mass flowing every second at the cold outlets, Cp is the capacity of a[illegible]t ambient during the test, T_c is the value of the air temperature through the cold outlets, and T_{in} is the temperature of the air entering the inlet vortex tube. The T_c and T_{in} values were obtained from the data measured. The following table presents the heat flow rate for cold outlets in ${}^{kJ}/_{s}$.

Table 20. Heat flow rate at cold outlets on natural cooling RHVT

Cold air mass fraction	Air pressure to inlet		
	0.5bar	1.0bar	1.5bar
30%	$0.221\,{}^{kJ}/_{s}$	$0.667\,{}^{kJ}/_{s}$	$1.415\,{}^{kJ}/_{s}$
40%	$0.326\,{}^{kJ}/_{s}$	$0.981\,{}^{kJ}/_{s}$	$2.043\,{}^{kJ}/_{s}$
50%	$0.352\,{}^{kJ}/_{s}$	$1.061\,{}^{kJ}/_{s}$	$2.248\,{}^{kJ}/_{s}$
60%	$0.385\,{}^{kJ}/_{s}$	$1.163\,{}^{kJ}/_{s}$	$2.462\,{}^{kJ}/_{s}$
70%	$0.403\,{}^{kJ}/_{s}$	$1.214\,{}^{kJ}/_{s}$	$2.562\,{}^{kJ}/_{s}$

Table 21. Heat flow rate at cold outlet on force cooling RHVT

Cold air mass fraction	Air pressure to inlet		
	0.5bar	1.0bar	1.5bar
30%	$0.265\,{}^{kJ}/_{s}$	$0.793\,{}^{kJ}/_{s}$	$1.656\,{}^{kJ}/_{s}$
40%	$0.375\,{}^{kJ}/_{s}$	$1.113\,{}^{kJ}/_{s}$	$2.293\,{}^{kJ}/_{s}$

50%	$0.455 {}^{kJ}/_{s}$	$1.341 {}^{kJ}/_{s}$	$2.750 {}^{kJ}/_{s}$
60%	$0.530 {}^{kJ}/_{s}$	$1.548 {}^{kJ}/_{s}$	$3.154 {}^{kJ}/_{s}$
70%	$0.582 {}^{kJ}/_{s}$	$1.659 {}^{kJ}/_{s}$	$3.280 {}^{kJ}/_{s}$

The amount of heat transferred by the vortex tube as a heating effect is denoted by $\dot{Q}_h$ and obtained using the following equation [13]:

$$\dot{Q}_h = \dot{m}_{outh}\, Cp\, (T_h - T_{in}) \quad (16)$$

The mass flow rate flowing at the hot outlet is denoted as $\dot{m}_{outh}$. The Cp value can be determined by reading the table while T is the temperature of the air coming into through the hot outlet. The following table presents the heat flow rate for hot outlets in ${}^{kJ}/_{s}$.

Table 22. Heat flow rate at hot outlets on natural cooling RHVT

Cold air mass fraction	Air pressure to inlet		
	0.5bar	1.0bar	1.5bar
30%	$0.222 {}^{kJ}/_{s}$	$0.547 {}^{kJ}/_{s}$	$1.037 {}^{kJ}/_{s}$
40%	$0.294 {}^{kJ}/_{s}$	$0.732 {}^{kJ}/_{s}$	$1.409 {}^{kJ}/_{s}$
50%	$0.379 {}^{kJ}/_{s}$	$1.023 {}^{kJ}/_{s}$	$2.041 {}^{kJ}/_{s}$
60%	$0.436 {}^{kJ}/_{s}$	$1.218 {}^{kJ}/_{s}$	$2.369 {}^{kJ}/_{s}$
70%	$0.228 {}^{kJ}/_{s}$	$0.667 {}^{kJ}/_{s}$	$1.377 {}^{kJ}/_{s}$

Table 23. Heat flow rate at hot outlets on force cooling RHVT

Cold air mass fraction	Air pressure to inlet		
	0.5bar	1.0bar	1.5bar
30%	$0.039 {}^{kJ}/_{s}$	$0.098 {}^{kJ}/_{s}$	$0.193 {}^{kJ}/_{s}$
40%	$0.085 {}^{kJ}/_{s}$	$0.240 {}^{kJ}/_{s}$	$0.501 {}^{kJ}/_{s}$
50%	$0.091 {}^{kJ}/_{s}$	$0.261 {}^{kJ}/_{s}$	$0.550 {}^{kJ}/_{s}$
60%	$0.087 {}^{kJ}/_{s}$	$0.259 {}^{kJ}/_{s}$	$0.550 {}^{kJ}/_{s}$
70%	$0.043 {}^{kJ}/_{s}$	$0.126 {}^{kJ}/_{s}$	$0.260 {}^{kJ}/_{s}$

The total compressed air power entering the inlet with ideal isothermal compression is as follows [3], [6], [7], [13]–[15]:

$$W = \dot{m}_{in}\, R\, T_{in}\, ln\, (P_{in}/P_{atm}) \quad (17)$$

From equation (17), $\dot{m}_{in}$ is the mass flow rate of air entering the inlet channel, R is the specific gas constant, T_{in} is the temperature of the air, and P_{in} is the air pressure entering the inlet channel. The

environmental air pressure is denoted by P_{atm}. The following table presents the total compressed air power entering the inlet in $^{kJ}/_{s}$.

Table 24. Total compressed air power entering the inlet

Air pressure to inlet		
0.5bar	1.0bar	1.5bar
$4.734\,^{kJ}/_{s}$	$14.928\,^{kJ}/_{s}$	$32.875\,^{kJ}/_{s}$

The coefficient of performance refrigeration (COP_{ref}) is a dimensionless number that measures the performance of a cooling heat pump engine when transferring heat from a cooled room [2], [12]. For a vortex tube, it is calculated as follows [2], [3], [12].

$$COP_{ref} = \dot{Q}_c/\dot{W} \tag{18}$$

The average COP_{ref} produced by vortex tube with natural cooling tube type is presented in table 25 while the vortex tube with forced cooling is shown in table 26. COP_{ref} numbers are dimensionless, therefore they are presented without units.

Table 25. COP_{ref} average air exits from the natural vortex tube cold cooling outlet

Cold air mass fraction	Air pressure to inlet		
	0.5bar	1.0bar	1.5bar
30%	0.047	0.046	0.044
40%	0.069	0.068	0.062
50%	0.074	0.074	0.070
60%	0.081	0.081	0.079
70%	0.085	0.083	0.083

Table 26. COP_{ref} the mean air coming out of the vortex cold tube forced cooling outlet

Cold air mass fraction	Air pressure to inlet		
	0.5bar	1.0bar	1.5bar
30%	0.056	0.054	0.050
40%	0.079	0.078	0.069
50%	0.096	0.093	0.087
60%	0.112	0.104	0.097
70%	0.123	0.111	0.100

The coefficient of performance heat pumps (COP_h) is a dimensionless number that measures the performance of a heat pump engine when transferring heat to a heated chamber[2], [12]. It was denoted as follows in the vortex tube [2], [3], [12].

$$COP_h = \dot{Q}_h/\dot{W} \tag{19}$$

Table 27 and 28 shows the average COP_h produced by vortex tubes with natural cooling tube types and vortex tubes with forced cooling respectively. There are no dimensions for COP_h numbers.

Table 27. COP_h mean air exit from the vortex tube and cold forced outlet

Cold air mass fraction	Air pressure to inlet		
	0.5bar	1.0bar	1.5bar
30%	0.047	0.037	0.036
40%	0.066	0.049	0.043
50%	0.080	0.068	0.062
60%	0.076	0.081	0.072
70%	0.039	0.043	0.042

Table 28. COP_h average air exits from the vortex tube cold forced outlet

Cold air mass fraction	Air pressure to inlet		
	0.5bar	1.0bar	1.5bar
30%	0.008	0.007	0.006
40%	0.018	0.016	0.015
50%	0.019	0.017	0.017
60%	0.018	0.017	0.017
70%	0.009	0.008	0.008

Experimental Design, Materials, and Methods

Data collection was carried out through experimental tests and processed mathematically. The RHVTs include types A with a natural cooling process and B with the forced cooling process, both on the surfaces of the tube. The RHVT used in types A and B was counter flow and the material used in all case was aluminium, except the cooling tube of B which used the black Teflon. The inlet diameter was 5mm, while the cold and hot tube had diameters and lengths 5mm and 40mm as well as 8mm and 105mm respectively. The diameter of the inlet and outlets of the cooling tube on type B RHVT was 8mm. However, an inner tube had a diameter of 25mm and a length of 75mm. Figure 1 shows the details of the RHVT specifications used.

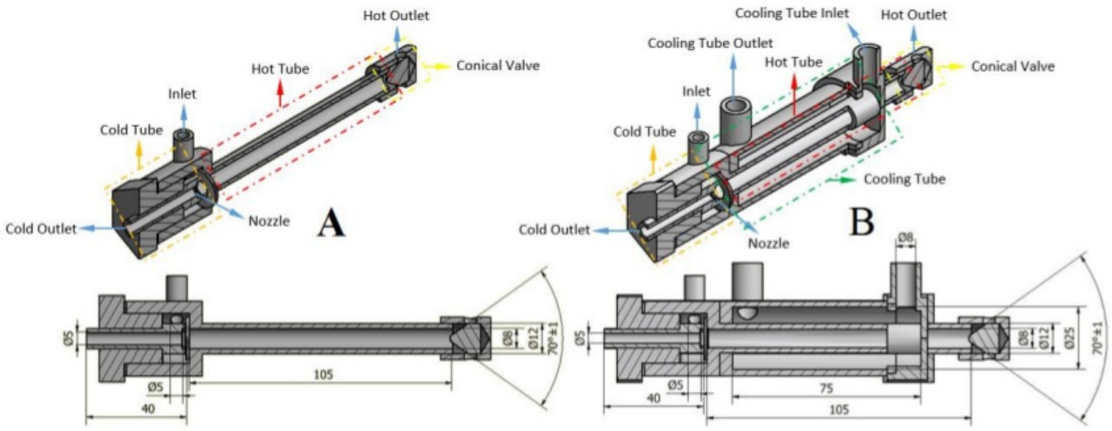

Fig. 1. Two types of vortex tubes, A and B

Types A and B had 4 nozzle holes and the air rotation is in the direction of the flow at the inlet. The nozzle hole is rectangular with dimensions of 1 × 2mm. The diameter of the round nozzles is 8mm with a thickness of 1.5mm. The detailed specifications of the RHVT contra flow nozzles used are presented in Figure 2.

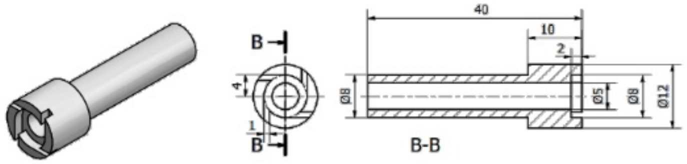

Fig. 2. Vortex tube specifications

Figure 3 shows a series of instruments used for experimental data collection. In the Left and right sides of the figure are Type A and B vortex tubes respectively. The temperature was set at 27°±0.1°C and data was recorded after 5 minutes of running.

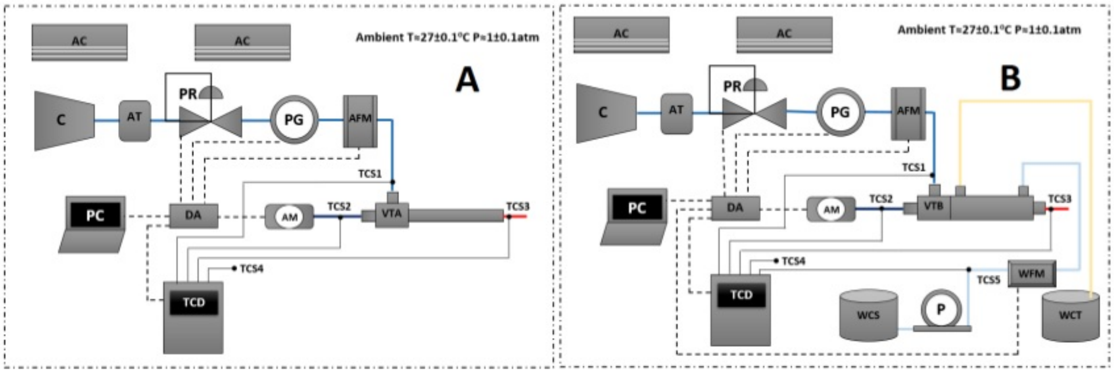

Fig. 3. Series of experimental tools for types A and B

Table 29. Experimental devices.

Initial	Name	Function	Symbol	Unit	Sensitivity values
AC	Air Conditioner	Room temperature controller	-	°Celsius	0.1
C	Compressor	Pressurized air supply	-	-	-
AT	Air Tank	Maintained air pressure so as not to drop, reduce humidity and return to room temperature	-	-	-
PR	Pressure Regulator	Sets the input air pressure	-	-	-
PG	Pressure Gauge	Measured inlet air pressure	P_{in}	bar	0.1
AFM	Flowmeter (air)	Measured airflow	$\dot{V}_{in}$	Liter/min	0.1
VTA / VTB	Vortex tube (type A and type B)	Test instrument	-	-	-
AM	Anemometer	Measured the speed of cold outlet air	$\vec{v}_{cn}$ and $\vec{v}_{cmax}$	meter/sec	0.1
TCD	Thermocouple Display	Measured hot and cold outlet air temperature	−	°Celsius	0.01
WCS	Water Container Storage	Saved water for cooling	-	-	-
WCT	Water Container Trash	Saved waste water for cooling	-	-	-
P	Pump	Pumped cooling water	-	-	-
WFM	Flowmeter (water)	Measured water discharge	$\dot{V}_{water}$	Liter/min	0.1
DA	Data Acquisition	Recorded measurement data	-	-	-
PC	Personal Computer	Processed observation data from each measuring instrument	-	-	-
TCS1	Thermocouple Sensor 1 (Type K)	Measured the temperature of the inlet air	T_{in}	°Celsius	-
TCS2	Thermocouple Sensor 2 (Type K)	Measured the temperature of the cold outlet air	T_c	°Celsius	-
TCS3	Thermocouple Sensor 3 (Type K)	Measured the temperature of the hot outlet air	T_h	°Celsius	-
TCS4	Thermocouple Sensor 4 (Type K)	Measured ambient air temperature	T	°Celsius	-
TCS5	Thermocouple Sensor 4 (Type K)	Measured the temperature of the cooling water air	-	°Celsius	-

Acknowledgments

This research is partially funded by the Indonesian Ministry of Research, Technology and Higher Education under WCU Program managed by Institut Teknologi Bandung.

References

[1] O. Aydin, B. Markal, and M. Avci, “A new vortex generator geometry for a counter-flow Ranque-Hilsch vortex tube,” *Appl. Therm. Eng.*, vol. 30, no. 16, pp. 2505–2511, 2010.

[2] A. Sarifudin, D. S. Wijayanto, and I. Widiastuti, “Parameters optimization of tube type, pressure, and mass fraction on vortex tube performance using the Taguchi method,” *Int. J. Heat Technol.*, vol. 37, no. 2, pp. 597–604, 2019.

[3] M. Attalla, H. Ahmed, M. S. Ahmed, and A. A. El-Wafa, “Experimental investigation for thermal performance of series and parallel Ranque-Hilsch vortex tube systems,” *Appl. Therm. Eng.*, vol. 123, pp. 327–339, 2017.

[4] V. Alekhin, V. Bianco, A. Khait, and A. Noskov, “Numerical investigation of a double-circuit Ranque-Hilsch vortex tube,” *Int. J. Therm. Sci.*, vol. 89, pp. 272–282, 2015.

[5] V. Kirmaci and H. Kaya, “Effects of working fluid, nozzle number, nozzle material and connection type on thermal performance of a Ranque–Hilsch vortex tube: A review,” *Int. J. Refrig.*, vol. 91, pp. 254–266, 2018.

[6] M. Attalla, H. Ahmed, M. Salem Ahmed, and A. Abo El- Wafa, “An experimental study of nozzle number on Ranque Hilsch counter-flow vortex tube,” *Exp. Therm. Fluid Sci.*, vol. 82, pp. 381–389, 2017.

[7] A. Kumar, Vivekanand, and S. Subudhi, “Cooling and dehumidification using vortex tube,” *Appl. Therm. Eng.*, vol. 122, pp. 181–193, 2017.

[8] S. Eiamsa-ard, K. Wongcharee, and P. Promvonge, “Experimental investigation on energy separation in a counter-flow Ranque-Hilsch vortex tube: Effect of cooling a hot tube,” *Int. Commun. Heat Mass Transf.*, vol. 37, no. 2, pp. 156–162, 2010.

[9] M. A. B. Yunus A. Çengel, *Thermodynamics: An Engineering Approach*, 5th ed. New York: McGraw-Hill, 2006.

[10] M. O. Hamdan, S. A. B. Al-Omari, and A. S. Oweimer, “Experimental study of vortex tube energy separation under different tube design,” *Exp. Therm. Fluid Sci.*, vol. 91, no. August 2017, pp. 306–311, 2018.

[11] S. E. Rafiee and M. M. Sadeghiazad, “Heat and mass transfer between cold and hot vortex cores inside Ranque-Hilsch vortex tube-optimization of hot tube length,” *Int. J. Heat Technol.*, vol. 34, no. 1, pp. 31–38, 2016.

[12] Y. A. . Cengel and J. M. . Cimbala, *Fluid Mechanics: Fundamentals and Applications*. 2014.

[13] R. Simo, “An air-standard cycle and a thermodynamic perspective on operational limits of Ranque – Hilsh or vortex tubes ` air traditionnel , aspects thermodynamiques et limites Cycle a du fonctionnement des tubes Ranque-Hilsch ou vortex,” vol. 33, pp. 765–773, 2010.

[14] K. D. Devade and A. T. Pise, “Effect of Mach number, valve angle and length to diameter ratio on thermal performance in flow of air through Ranque Hilsch vortex tube,” *Heat Mass Transf. und Stoffuebertragung*, vol. 53, no. 1, pp. 161–168, 2017.

[15] S. Subudhi and M. Sen, “Review of Ranque-Hilsch vortex tube experiments using air,” *Renew. Sustain. Energy Rev.*, vol. 52, pp. 172–178, 2015.

DATASET OF COMPREHENSIVE THERMAL PERFORMANCE ON COOLING THE HOT TUBE SURFACES OF VORTEX TUBE AT DIFFERENT PRESSURE AND FRACTION

ORIGINALITY REPORT

10%	4%	5%	7%
SIMILARITY INDEX	INTERNET SOURCES	PUBLICATIONS	STUDENT PAPERS

PRIMARY SOURCES

1	Submitted to Universitas Sebelas Maret<br>Student Paper	2%
2	iieta.org<br>Internet Source	1%
3	Valentina Fortunato, Andres Giraldo, Mehdi Rouabah, Rabia Nacereddine, Michel Delanaye, Alessandro Parente. "Experimental and Numerical Investigation of a MILD Combustion Chamber for Micro Gas Turbine Applications", Energies, 2018<br>Publication	1%
4	www.iieta.org<br>Internet Source	1%
5	Rafiee, S.E., and M.M. Sadeghiazad. "Experimental and 3D CFD investigation on heat transfer and energy separation inside a counter flow vortex tube using different shapes of hot control valves", Applied Thermal	1%

Engineering, 2017.
Publication

6	www.mdpi.com<br>Internet Source	1%
7	Ouadha, A., M. Baghdad, and Y. Addad. "Effects of variable thermophysical properties on flow and energy separation in a vortex tube", International Journal of Refrigeration, 2013.<br>Publication	<1%
8	Subudhi, Sudhakar, and Mihir Sen. "Review of Ranque–Hilsch vortex tube experiments using air", Renewable and Sustainable Energy Reviews, 2015.<br>Publication	<1%
9	archive.org<br>Internet Source	<1%
10	Submitted to Arizona State University<br>Student Paper	<1%
11	Submitted to University of Bradford<br>Student Paper	<1%
12	timtailieu.vn<br>Internet Source	<1%
13	Submitted to Universiti Putra Malaysia<br>Student Paper	<1%
14	Submitted to International Islamic University	

Malaysia
Student Paper | <1%

15 ijesmr.com
Internet Source | <1%

16 pt.scribd.com
Internet Source | <1%

17 Submitted to UC, Boulder
Student Paper | <1%

18 Submitted to University Of Tasmania
Student Paper | <1%

19 eds.yildiz.edu.tr
Internet Source | <1%

20 Submitted to The College of New Jersey
Student Paper | <1%

21 Wisnoe, Wirachman, Nurhasanah Ismail, Muhammad Fairuz Remeli, and Muhamad Fairul Zakaria. "Experimental investigation on the effect of conical valve shape and swirl generator to the performance of Ranque-Hilsch Vortex Tube", 2013 IEEE Business Engineering and Industrial Applications Colloquium (BEIAC), 2013.
Publication | <1%

22 Hamdan, Mohammad O., Ahmed Alawar, Emad Elnajjar, and Waseem Siddique. "Feasibility of | <1%

Vortex Tube Air-Conditioning System", ASME/JSME 2011 8th Thermal Engineering Joint Conference, 2011.
Publication

23	Alfan Sarifudin, Danar Wijayanto, Indah Widiastuti. "Parameters Optimization of Tube Type, Pressure, and Mass Fraction on Vortex Tube Performance Using the Taguchi Method", International Journal of Heat and Technology, 2019<br>Publication	<1%
24	Kumar, G. Suresh, G. Padmanabhan, and B. Dattatreya Sarma. "Optimizing the Temperature of Hot outlet Air of Vortex Tube using Taguchi Method.", Procedia Engineering, 2014.<br>Publication	<1%
25	Submitted to University of Hertfordshire<br>Student Paper	<1%

Exclude quotes	Off	Exclude matches	Off
Exclude bibliography	On		

DATASET OF COMPREHENSIVE THERMAL PERFORMANCE ON COOLING THE HOT TUBE SURFACES OF VORTEX TUBE AT DIFFERENT PRESSURE AND FRACTION

GRADEMARK REPORT

FINAL GRADE

/0

GENERAL COMMENTS

Instructor

PAGE 1

PAGE 2

PAGE 3

PAGE 4

PAGE 5

PAGE 6

PAGE 7

PAGE 8

PAGE 9

PAGE 10

PAGE 11

PAGE 12

PAGE 13

PAGE 14

PAGE 15

PAGE 16
